# Assessing healthcare capacity crisis preparedness: development of an evaluation tool by a Canadian health authority

**DOI:** 10.3389/fpubh.2023.1231738

**Published:** 2023-10-10

**Authors:** Breitner Gomes Chaves, Hassane Alami, Brigitte Sonier-Ferguson, Erika N. Dugas

**Affiliations:** ^1^Vitalité Health Network, Dr. Georges-L.-Dumont University Hospital Centre, Moncton, NB, Canada; ^2^École de Santé Publique, Université de Montréal, Montreal, QC, Canada

**Keywords:** health evaluation, crise management, COVID-19 pandemic, pandemic management, tool development

## Abstract

**Introduction:**

The COVID-19 pandemic presented health systems across the globe with unparalleled socio-political, ethical, scientific, and economic challenges. Despite the necessity for a unified, innovative, and effective response, many jurisdictions were unprepared to such a profound health crisis. This study aims to outline the creation of an evaluative tool designed to measure and evaluate the Vitalité Health Network’s (New Brunswick, Canada) ability to manage health crises.

**Methods:**

The methodology of this work was carried out in four stages: (1) construction of an evaluative framework; (2) validation of the framework; (3) construction of the evaluative tool for the Health Authority; and (4) evaluation of the capacity to manage a health crisis.

**Results:**

The resulting evaluative tool incorporated 8 dimensions, 74 strategies, and 109 observable elements. The dimensions included: (1) clinical care management; (2) infection prevention and control; (3) governance and leadership; (4) human and logistic resources; (5) communication and technologies; (6) health research; (7) ethics and values; and (8) training. A Canadian Health Authority implemented the tool to support its future preparedness.

**Conclusion:**

This study introduces a methodological strategy adopted by a Canadian health authority to evaluate its capacity in managing health crises. Notably, this study marks the first instance where a Canadian health authority has created a tool for emergency healthcare management, informed by literature in the field and their direct experience from handling the SARS-CoV-2 pandemic.

## Background

The World Health Organization (WHO) announced on March 11, 2020, that the COVID-19 outbreak had reached pandemic status. Health systems worldwide grappled to mobilize, organize, and deploy resources rapidly to minimize the disease’s devastating public health consequences (1, 2). In addition to the devastating mortality rates, the pandemic worsened existing chronic medical conditions and exacerbated health inequalities, particularly among vulnerable and disadvantaged populations (3, 4). Some experts have coined the term “syndemic” to describe the combined health, socio-political and socioeconomic effects of the pandemic (5). Importantly, the unprecedented demand and pression on health and social services associated with the COVID-19 pandemic revealed vulnerabilities and lack of preparedness of modern health systems worldwide (6). In 2020, only 57% of countries were ready to prevent, detect, and control a pandemic such as COVID-19 (7). However, The COVID-19 pandemic revealed that even countries with strong healthcare systems were not immune to the widespread challenges presented by the crisis, highlighting its intricate nature (8, 9).

In Canada, despite ongoing efforts to mitigate the consequences of the virus, the impact of COVID-19 on health system and economy continues to be felt (10, 11). One of the most significant lessons from this crisis was the need for a coordinated, multilevel, intersectoral and innovative response that encompasses a range of convergent and effective actions (12, 13). Different Canadian health organizations and systems have developed protocols, actions plans and frameworks to facilitate the response to the COVID-19 pandemic and other sanitary crises (14). However, the strategies designed were not always consistent and several jurisdictions struggled to ensure convergent, effective, and coordinated actions adapted to its own context (15–17).

In this context, the provincial New-Brunswick government quickly developed and implemented strict guidelines and strategies to fight COVID-19 crises (Box 1). These included early implementation of border closures, school shutdowns, and mandatory quarantine periods (18).

From the beginning, Vitalité Health Network (VHN)‘s pandemic response was framed using a health care approach focussed on four cornerstones resilience capacities: monitor, anticipate, respond, and learn while maintaining quality of usual care (19, 20). Building on a culture of collaboration, organizational capacity and resilience developed during the COVID-19 pandemic, VHN implemented rapidly changing adaptative strategies.

The scientific literature has proposed numerous frameworks to assist health systems in managing health crises and their evaluation processes (14, 21–26). Some of these are specifically tailored to the challenges posed by the COVID-19 pandemic. Nevertheless, several of these frameworks exhibit notable limitations. They often possess a complexity that challenges practical implementation within daily health organization operations or lack sufficient context descriptions, making them less adaptable to the specific needs of health authorities. Therefore, it was crucial to develop an adaptable tool to support the adequate preparedness level of the health authority network to respond to any potential health crises in the future. The rationale behind the development of this tool stems from our understanding that health systems are inherently distinct, with numerous contextual factors interplaying with their core elements. Hence, while VHN drew inspiration from existing tools in the scientific literature, we chose to construct our own custom evaluation instrument. This tool’s legitimacy is further bolstered by the insights of our experts who have firsthand experience with the challenges posed by the COVID-19 crisis. Following a literature review, but also our networks of experts and researchers in the field in Canada, we have not found any evidence of other evaluative tools developed and evaluated by other Canadian health authorities that both encapsulate the learnings acquired during the pandemic and concurrently develop a tool for internal self-assessment following a timeline in a fundamentally organic (in-house) manner.

Considering the lessons learned from the COVID-19 crisis, the aim of this article was to outline the process utilized to evaluate the VHN’s capacity for managing current COVID-19 pandemic as well as a possible future health crisis. Specific objectives were to: (1) develop a health crisis capacity tool adapted to the local context; and, with the network’s tool, (2) evaluate VHN’s current capacity to manage a health crisis.

BOX 1 **Overview of the organization and governance of New Brunswick’s healthcare system.**

Canada, as a federation, comprises 10 provinces and three territories, each possessing substantial autonomy over their policies. Health matters fall primarily under the purview of the provinces, but the federal government subsidizes provincial health initiatives, contingent on adherence to the Canada Health Act (13). The health care system of New Brunswick, the largest of Canada’s three Maritime provinces (population of 776,827), is overseen by the New Brunswick Department of Health. Health services are delivered through two health authorities, each divided in 4 local zones. Horizon Health Network delivers care to southern and central-west New-Brunswick (predominantly English-speaking population) and Vitalité Health Network (VHN) delivers care to northern and southeastern New-Brunswick (predominantly French-speaking population) Encompassing a network of approximately 70 diverse sites and facilities, inclusive of 11 hospitals, Vitalité Health Network attends to the healthcare demands of over 136,000 patients on an annual basis. It provides a comprehensive array of services spanning primary, secondary, and tertiary healthcare levels, ensuring a full continuum of care. VHN is supported by a team of approximately 7,400 dedicated professionals, which includes doctors, nurses, and an array of specialized healthcare providers. Ambulance New Brunswick (ANB) operates as the province’s ambulance service and is a part of the public entity, EM/ANB Inc., which falls under the New Brunswick Companies Act and reports to the Minister of Health. All management duties for land and air ambulances are entrusted to Medavie Health Services New Brunswick, a branch of Medavie Health Services which is independent of VHR authority. To date (Juillet 2023), New-Brunswick reported 91,265 confirmed cases of COVID-19 and 928 deaths attributed to the virus (included in the total of 4.7 million confirmed cases and 53,000 deaths reported in Canada).

## Methodology

In April 2022, the research and health evaluation sector of VHN was mandated by the leadership team to gather and systematize the knowledge and experience acquired by the Network during the COVID-19 pandemic. To execute this mandate, we sought to develop a tool to measure the Networks’ current and future capacity to manage health crises. This work was carried out in four steps: (1) construction of an evaluative framework; (2) validation of the evaluative framework; (3) development of the evaluative tool; and (4) evaluation of the organizational health crisis management capacity. The following sections detail the methodology for each step.

### Step I: construction of the evaluative framework

**Figure 1:**
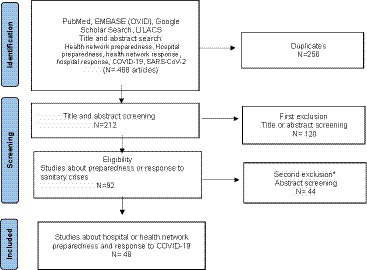
Literature review path. *Articles that were not directly related to the COVID-19 pandemic or focused on a ministerial level of analysis (political level).

A literature review in PubMed, EMBASE (OVID), Google Scholar, and LILACS was undertaken in June 2022 to identify English, French, Spanish, and Portuguese studies published between 2019 and 2022 that detailed health authorities or hospitals’ responses and strategies to manage a health crisis. The following keywords were applied in many Boolean operators’ combinations for titles and abstracts: “hospital preparedness”; “hospital response”; “health network preparedness”; “health network response”; “COVID-19″; and “SARS-Cov-2″. These searches yielded a total of 212 titles after removal of duplicates. Among them, forty-eight articles were retained for review (Figure 1).

An evaluation team of three professionals (a public health evaluation researcher, a performance/planning director, and a risk management director) was responsible for the entire research and analysis process. In this phase, we established three criteria for selecting the key strategies to be incorporated into the developing tool: (1) the extent of the VHN’s experience in implementing the strategy, (2) the potential impact of the strategy within the VHN context, and (3) the practicality and feasibility of executing the strategy. Including the director of performance/planning and the director of risk management in this process was crucial to ensure a first validation of the pertinence and feasibility of the selected strategies. The team discerned that the 85 distinct strategies they identified could be organized into eight core dimensions aligned with the organizational structure and governance of VHN. It would simplify the construction of the evaluative tool by dimensions or categories and facilitating the evaluation process. Thus, the 85 strategies were categorized under eight broad dimensions, reflecting, and considering the corporate governance structure of the VHN: (1) clinical care management; (2) human and logistic resources; (3) governance and leadership; (4) communication and technologies; (5) infection control and prevention; (6) health research; (7) ethics and values; and (8) training. The Supplementary material 3 highlights all selected articles.

### Step II: validation of the evaluative framework

Organizational research suggests that contextual determinants can influence health interventions or their components, potentially leading to unintended outcomes (27, 28). Therefore, a validation step was included in the methodological approach not only to confirm the importance of the strategies identified in the previous stage, but also their feasibility within the unique context of VHN. In other words, the results of the literature review (step 1) were further supplemented with expert opinion and practical advice.

In step 2, the evaluation team identified and reached out to fifty of VHN’s organizational experts via email (identified as key individuals for each dimension by both the crisis management director and the planning director), requesting them to assess each strategy according to their specific expertise. The selection criteria were based on whether the manager or clinician primarily implemented the strategies during the COVID-19 crisis, or if they were directly associated with the dimensions examined within the organization. Supplementary material 1 details the number and profile of experts who took part in this validation stage for each dimension.

Experts were asked to evaluate online the level of importance of each strategy in responding to a health crisis (i.e., for the entire network and not for a specific hospital), specifically the COVID-19 pandemic, within the context of VHN. Using a 4-point Likert scale, experts rated each strategy from “not at all important” to “extremely important.” Only strategies rated as “very important” or “extremely important” by all experts were retained (*n* = 74/85). Eleven strategies, on average 1 or 2 per dimension, were deemed as “not at all important” or “not important” by the experts within their respective dimensions. As a result, these strategies (11 out of 85) were excluded from the emerging framework. No specific statistical treatment was required for this analysis. According to implementation science and organizational research, establishing consensus and a shared vision among stakeholders can enhance the likelihood of successful complex health interventions (29–31). Therefore, we aimed to foster agreement on the pertinence of all strategies with the objective of promoting adherence to the project, improve its credibility, and facilitate the implementation of future recommendations.

### Step III: development of the evaluative tool

Building upon the final evaluative framework of 74 retained strategies across 8 dimensions, we encountered two significant challenges while developing the evaluative tool to assess the VHN’s health crisis management capacity. The first challenge involved finding an objective method to identify the presence or absence of the strategies within the organization. To address this, the evaluation team developed “observable elements” for each retained strategy to objectively identify the presence or absence (or the degree of implementation) of these 74 retained strategies. By creating observable elements for each strategy, we aimed to guide potential evaluators in finding evidence of strategies implementation toward the organization. The pivotal question used to generate each observable factor tied to each strategy was: “What steps can we take to observe that this specific strategy is executed or not in our organization?” For instance, when considering the strategy “S2.4 Ability to manage oxygen shortage,” the observable element indicating the presence of this strategy in our network was the existence of a contingency plan to handle such a scenario. Doing this for all strategies, a total of 109 observable elements were developed to estimate the presence or the degree of implementation of the 74 validated strategies identified in the previous phase. Supplementary material 2 provides a detailed breakdown of the observable elements associated with each strategy. Essentially, it delineates how the implementation status of each strategy was determined within the organization during the evaluation phase.

A second challenge emerges from the varying importance assigned to each dimension that contribute to the final indicator evaluating the network’s ability to handle a health crisis. According to WHO guidelines, health systems should reflect on the weight of the chosen dimension in their effort to improve their capacity to respond to health crises, considering their economic, social and political context (32). This indicates that certain dimensions hold greater “weight” than others when determining the capacity to manage a health crisis, such as the COVID-19 pandemic, as per local stakeholders. The weight (adjustment factor) assigned to each dimension tool was determined through collaboration among the crisis management director, the planning director, and a public health expert. The criteria used to assign weight were as follows: (1) The general impact of the dimension on local health crisis management; (2) The degree of influence that each dimension has on the others; (3) The potential risks posed to patients and professionals if a dimension is poorly managed. For instance, the work group determined that the “prevention and infection control dimension” should carry more weight than the “training dimension” within the tool. Their objective was to ensure a fair and accurate assessment, considering the local context. More details can be found in Supplementary material 2.

Thereafter, each of the 74 retained strategies was assigned a score to indicate its presence to allow the calculation of the score for each dimension. The evaluation tool allowed for the generation of eight indicators, each representing the status or degree of implementation related to a specific dimension. Moreover, the final global organizational indicator, the health crisis management capacity (HCMC), was derived based on the individual values of each dimension, considering their respective importance in the organization’s capacity to manage health crises.

### Step IV: evaluation of the organizational health crisis management capacity

In January 2023, the evaluative tool was implemented. The evaluation process was as follows:

1. Evaluators identify managers and clinicians associated with the eight proposed dimensions and schedule interviews with them.
2. During these interviews, managers are prompted to provide tangible evidence illustrating the extent of the evaluated strategies’ implementation.
3. If the strategies align with the criteria established by the predefined observable elements, a decision is made to assign one (1) point (for more detailed information, refer to Supplementary material 2).
4. A unique score is then calculated for each dimension, a score between 0 and 100%, reflecting the proportion of achievable points obtained (detailed in Supplementary material 2)
5. Lastly, scores for each dimension were incorporated into a formula (a simple sum from each dimension detailed in Supplementary material 2) to determine the final indicator (HCMC).

The establishment of observable elements in the previous step was crucial in optimizing and streamlining the evaluators’ work. These elements acted as a guide, indicating what to look for to demonstrate the presence of the organization’s strategies. Using the proportion of strategies implemented, the group determined a final score for each dimension (a score between 0 and 100%, reflecting the proportion of achievable points obtained). Finally, the final indicator, HCMC, was calculated, considering the weight of each dimension (adjustment factor) established in the previous phases.

## Results

In this study, the evaluative tool was formulated by incorporating multiple strategies. The final evaluation tool includes 8 dimensions, 74 strategies, and 109 observable elements (see Supplementary material 2). Table 1 presents the characteristics of strategies and their relevance in building the evaluative tool.

**Table 1:** Evaluative tool dimension description and relevance.

Dimension	Characteristics of the strategies	Relevance
Clinical care management	Strategies related to clinical response capacity, such as clinical protocols, availability of complementary tests, and integration and availability of clinical data for quick decision-making.	Due to the overload of health services, the importance of clinical protocols, bed management, efficient laboratory support, and clinical data integration becomes paramount (33, 34).
Human and Logistic Resources	This dimension encompasses strategies related to effectively managing and optimizing human resources and supply chain in a crisis scenario.	Resource planning and preparedness are necessary to cope with the pressure on healthcare networks in the context of health crises. A rapid and effective response is critical to health system responsiveness (35, 36).
Governance and leadership	This dimension presents strategies related to strategic crisis management, governance, and decision-making across the organization	In a crisis scenario, effective leadership and governance models are crucial for optimizing responses to a health crisis. Leadership plays a critical role in shaping the collective response, while governance models ensure the smooth functioning of the healthcare networks (37, 38).
Communication and technologies	We highlight strategies that facilitate both internal communication within the organization and the dissemination of critical information to stakeholders.	Effective communication can have a significant impact on promoting health behavior change among stakeholders, fostering organizational cohesion, and advocating for excellence in healthcare (39).
Infection control and prevention	There are several strategies that fall under the umbrella of infection prevention and control, which aim to prevent or mitigate the spread of infectious diseases across the healthcare network.	These strategies can save lives and reduce health system burdens (40, 41)
Health Research	Strategies related to medical research and its ethical implications, in addition to the timely transfer of knowledge.	Given the scenario of high uncertainty and limited information, the ability to mobilize researchers from diverse disciplines to develop or adapt context-specific strategies can support organizational responsiveness (42, 43).
Ethic and values	This dimension involves strategies aimed at preventing or mitigating the adverse effects of crisis response actions on vulnerable populations.	Health crises pose a major challenge for health systems as they must not only address the immediate crisis but also prevent or mitigate any negative consequences of their actions (44, 45).
Training	Strategies aimed at developing the necessary competencies for network professionals to effectively respond during a health crisis	Education and training promote resilience in crisis situations and prepare for an uncertain future. The learning and evaluation cycle fosters improvements and innovation for a better response to health crises (33, 35).

Table 2 displays the scores for each dimension. The table is organized into five columns. In our evaluation, we have structured our findings into five columns for clarity. The first column, “Dimension,” categorizes the eight tool’s dimensions. The second, “Score,” provides raw scores for each dimension, stemming from the evaluation of strategies in that specific area. For instance, “clinical care management” garnered a score of 17.5, which signifies the strategies’ performance during the evaluation phase.

**Table 2:** Score and final indicator for each dimension, January 2023.

Dimension (1)	Score (2)	% (0 to 100%) (3)	Adjustment factor (4)	Final dimension Score (5)
Clinical care management	17.5	79.6	0.2	15.92
Human and logistic resources	21	75	0.2	15
Governance and leadership	16	88.9	0.1	8.89
Communication and technologies	14	87.5	0.1	8.75
Infection control and prevention	26	81.25	0.2	16.25
Health research	5.5	55	0.05	2.75
Ethic and values	6.5	65	0.05	3.25
Training	9	75	0.1	7.5

The third column represents the proportion each strategy occupies within its domain. To illustrate, the 17.5 score in “clinical care management” amounts to 79.6% of the highest possible score for that category (100%). This percentage representation holds true for every other dimension, with comprehensive details found in Supplementary material 2.

Next, the “Adjustment factor” column offers an insight into the significance or weight of each dimension. Essentially, it’s an adjustment factor that’s further elucidated in the third step of our methodology. The final column, “Final Dimension Score,” showcases the adjusted score by multiplying the raw score with its corresponding adjustment factor.

As of January 2023, the aggregate score across all evaluated dimensions amounted to 78.3%. This consolidated figure serves as an indicator, benchmarking our organizational aptitude in addressing health crises, contingent upon the strategies we have presently employed. It is important to note that the score mentioned corresponds to dimensions at the organizational level, rather than directly to individual units within the organization. This assessment is conducted systematically and may involve the implementation of intersectoral strategies.

## Discussion

This study outlines an initiative of a Canadian Health Authority to develop an evaluation tool that leverages its experience accumulated during the COVID-19 pandemic. It provides an original contribution to the literature on the management of health crises, showcasing how a Health Authority can employ a systematic approach for self-review and enhanced readiness, grounded in their learnings from the COVID-19 crisis. Based on the scientific literature (13–15, 21, 23, 24, 26, 33, 36, 46) and VHN’s own experience, VHN synthesized their organizational learning into eight core dimensions and 74 strategies over the course of the evaluation process.

By January 2023, nearly 3 years into the ongoing pandemic, VHN had successfully established an overarching indicator to assess its preparedness in managing health crises, predicated on its strategic implementations. Using the new evaluative tool, this indicator was determined to be at 78%. The “governance and leadership” dimension received the highest evaluation, with 88.9% of strategies implemented. It was closely followed by the “Communication and technologies” dimension at 87.5%, and the “Infection control and prevention” dimension at 81.25%.

Health authorities worldwide faced significant challenges beyond the “acute phases” during the COVID-19 pandemic – changing the healthcare landscape forever. Embedding Learning Health Systems into healthcare and continued coordinated, multilevel, and innovative responses are imperative, now more than ever, to remain up to date preventive, early detection, and response functions (13, 47).

Palagyi et al. (33) introduced a conceptual framework through a narrative synthesis that encompasses six core dimensions. Four of these focus on material resources and structures, while the other two emphasize human and institutional relationships, values, and norms. A pivotal contribution of their work highlighted the critical role of system ‘software’—like “governance and trust”—in effectively responding to health crises. Despite the innovative features of the Palagyi’s model, its expansive scope can pose challenges for service managers seeking to employ it as an evaluation tool. The evaluation framework suggested by VHN integrates elements from both dimensions, highlighting the significance of organizational governance coupled with clear and efficient communication.

Similarly, Adelaja et al. (21) drew from the National Health System (NHS) experience with COVID-19 to propose the Comprehensive Hospital Agile Preparedness (CHAPs) tool. This tool addresses pandemic readiness through six interconnected domains: workforce; infrastructure; supplies and equipment; service reconfiguration; data and information technology; and communications. Nevertheless, the authors did not develop a formalized tool to facilitate a systematic evaluation process. Additionally, the model does not account for the management of ethical implications tied to the strategies implemented.

Adam (48) introduce the interesting concept of surge capacity as “*the ability to obtain adequate staff, supplies and equipment, structures and systems to provide sufficient care to meet immediate needs of an influx of patients following a large-scale incident or disaster*” (48) This description embodies the theoretical frameworks associated with the four primary construct of hospital surge capacity: (1) staff or human resources; (2) stuff or equipment and supplies; (3) structure or physical space; and (4) systems that include integrated management policies and processes (49, 50). Our tool incorporates these components across various dimensions (ex. Human and Logistic Resources dimension).

Specifically, entities like the Pan American Health Organization (PAHO) (51) and the Centers for Disease Control and Prevention (CDC) (52) have crafted tools and checklists tailored for hospital assessments. In a more recent development, Seyedin et al. offered a checklist aimed at gauging preparedness across hospital settings. This thorough checklist was anchored on two pivotal pillars measures at national and measures at hospital level. At the national spectrum, there are three main components supervised by the health ministry (legal, referral and coordination), whereas measures specific to hospitals are branched into 24 separate classifications. However, this checklist has not yet been tested (37).

The VHN-developed tool has several merits: (1) *Practical Orientation:* The tool is grounded in its actionable and user-centric design. It’s been validated by a panel of experts, all of whom were directly engaged during local COVID-19 crises. (2) *Real-world Testing:* The tool was tested in a process of assessing the capacity to manage a health crisis by a Canadian health Authority (i.e., VHN). Moreover, organizational leadership teams integrated this tool to track the advancement and implementation of selected strategies across various areas. (3) *Academic Complement:* The tool serves as a complement to other frameworks in the literature, which often tend to be broader, sometimes abstract, and challenging to convert into actionable steps (4) *Ethical Component:* A standout feature is the tool’s emphasis on ethical reflections, particularly concerning at-risk populations. The goal is to address and potentially reduce health disparities that can emerge during health crises. This is especially pertinent since past research has highlighted the adverse effects of organizational decisions during the COVID-19 pandemic on individuals with chronic conditions, mental health disease, or those awaiting surgeries and other medical interventions (44, 53, 54).

Three key practical benefits to VHN were identified during the tool implementation. Firstly, it facilitates a continuous improvement of operational readiness for health crises by enabling the organization to identify its strengths and weaknesses at all levels and communicate them to all employees. Secondly, it allows for the comparison and tracking of responsiveness over time, helping to allocate resources where they are most needed to improve the overall response. Third, the framework provides a rapid and comprehensive overview of all critical dimensions, facilitating information dissemination and informed decision-making.

Although the evaluative tool was designed specifically for VHN, the methodology developed could be adapted and considered by other local health managers (Department of Health and Health authorities). This would facilitate integration and action coordination at the provincial level, potentially enhancing the overall effectiveness and responsiveness in New Brunswick, Canada.

In the second phase of this tool’s development, the inclusion of a broader array of experts served dual purposes. Primarily, it enhanced our methodology. Concurrently, it fostered a sense of inclusion among the experts, familiarizing them with the tool from its inception. Beyond serving as an additional validation layer, this stage facilitated introspection, enabling the experts to assimilate and reflect upon their insights – a pivotal, albeit indirect, outcome of this evaluative trajectory.

Also, the findings from the evaluation process will be circulated across the organization. The Crisis Management Department will spearhead the annual review of the evaluated dimensions and track their progress. The objective is to not only enhance the areas with suboptimal scores but also to facilitate collaboration among various sectors when specific strategies necessitate interdepartmental coordination.

In conclusion, this tool development sets an interesting foundation for upcoming initiatives aiming to develop instruments that assist health authorities in decision-making and real-world handling of health emergencies. However, to ensure that the evaluation tool remains abreast of new scientific evidence and the local reality (Network strategies, environmental changes, etc.), we recommend for VHN reviewing the tool at least yearly. Future studies should focus on understanding the weight (real impact) of each chosen dimension (and strategies) during different stages and contexts of a health emergency.

## Limitations

This study presents some limitations that must be acknowledged. Firstly, the evaluative tool was developed and tailored according to VHN’s context, and therefore, cannot be replicated to other health authorities without appropriate adaptations and/or adjustments. It is noteworthy that strategies deemed essential by local experts may not be feasible or effective in other settings.

Secondly, select critical dimensions of health emergency management, such as prevention and monitoring of emerging health threats, were not considered due to the limited mandate of the local health authority. While some strategies might overlap, the tool is not specifically tailored to address scenarios involving Mass Casualty Incidents (MCI). Such scenarios typically necessitate the creation of distinct contingency protocols across various levels, including political frameworks, health networks, hospitals, and communities.

Nevertheless, the leaders of VHN are actively working toward expanding the methodology applied in this study to the provincial level, enabling the development of a comprehensive and systematic plan for the entire provincial health system. Finally, we recommend that organizations’ local experts adjust the weighting of each dimension according to their own context and criteria.

## Conclusion

Regional health authorities must remain resilient and develop and deploy adaptative strategies to be prepared to fight the present and future pandemics. In adherence to an organizational directive, VHN established an internal methodology that systematically collates insights and strategies gathered during the COVID-19 pandemic.

Using the outlined methodology, the tool developed not only provides a clearer organizational perspective on the simultaneous deployment of various, but also facilitate longitudinal surveillance. Moreover, the methodological path can inspire other Health Authorities to methodically document and disseminate the insights gained during the COVID-19 pandemic. Finally, another aim of this paper is to encourage other health authorities to undertake self-evaluation exercises, facilitating broader comparisons between Canadian provinces and regions globally.

## Data availability statement

The original contributions presented in the study are included in the article/Supplementary material, further inquiries can be directed to the corresponding author.

## Author contributions

BG reviewed the literature, developed the evaluation tool, conducted data analyses, and wrote the first draft of the article. BS-F, HA, and ED reviewed the literature and wrote sections of the article. All authors contributed to conceptualization of the study and the analytic plan, interpreted the results, reviewed the article critically, approved the final version, and responsible for the reported research.
