# Supplementary material for: Assessing healthcare capacity crisis preparedness: development of an evaluation tool by a Canadian health authority
Source: Front Public Health. 2023 Oct 10;11:1231738. doi: 10.3389/fpubh.2023.1231738 (PMC10594116; doi:10.3389/fpubh.2023.1231738)
Supplement: Supplementary file 1 [file Table_1.docx]

**Supplement 1: Profile of experts that participated in the validation stage**

| **Dimension** | **Participants (N)** | **General profile** |
| --- | --- | --- |
| Clinical care management | 14 | Nurses, doctors, other health professional, sector managers |
| Infection control and prevention | 11 | Specialized Nurses and sector managers |
| Governance and leadership | 3 | Head directors |
| Logistic Resources | 8 | sector managers |
| Human Resources | 4 | sector managers |
| Communication and technologies | 2 | Communication professionals |
| Medical research and technologies | 3 | Researchers and sector managers |
| Ethic and values | 2 | Members of the local research ethics committee |
| Training | 3 | sector managers |
| **Total** | **50** |  |
