# Supplementary material for: Assessing healthcare capacity crisis preparedness: development of an evaluation tool by a Canadian health authority
Source: Front Public Health. 2023 Oct 10;11:1231738. doi: 10.3389/fpubh.2023.1231738 (PMC10594116; doi:10.3389/fpubh.2023.1231738)
Supplement: Supplementary file 2 [file Table_3.docx]

**Supplement 3: 48 selected articles**

| **ARTICLE** | **AUTHORS** | **Highlights** | **YEAR** |
| --- | --- | --- | --- |
| A Conceptual and Adaptable Approach to Hospital Preparedness for Acute Surge Events Due to Emerging Infectious Diseases | Anesi, George L.  Lynch, Ylinne  Evans, Laur  (12) | Provide a framework for considering key resources during an acute surge event—the “four Ss” of preparedness: space (beds), staff (clinicians and operations), stuff (physical equipment), and system (coordination) | 2020 |
| Hospital disaster preparedness in Iranian province: A cross-sectional study using a standard tool | Beyramijam, M.  Khankeh, H.  Shahabi-Rabori, M. A.  Aminizadeh, M.  Sheikhbardsiri, H.  (24) | In this research, 15 hospitals took part Most of these hospitals (53.33%) demonstrated a moderate level of preparedness, while 26.66% showed good preparedness, and 13.33% exhibited a low level. | 2021 |
| Rapid response infrastructure for pandemic preparedness in a tertiary care hospital: lessons learned from the COVID-19 outbreak in Cologne, Germany, February to March 2020 | Augustin, Max  Schommers, Philipp  Suárez, Isabelle  Koehler, Philipp  Gruell, Henning  Klein, Florian  Maurer, Christian  Et al  (23) | The article outlines the establishment of a COVID-19 rapid response system (CRRI) to relieve the ER from a critically high patient load and to maintain its functionality. | 2020 |
| A comprehensive hospital agile preparedness (CHAPs) tool for pandemic preparedness, based on the COVID-19 experience | Adelaja, Ini  Sayma, Meelad  Walton, Henry  McLachlan, Greta  de Boisanger, James  Bartlett-Pestell, Sam  Et al  (21) | The comprehensive hospital agile preparedness (CHAPs) tool provides clinical planners with six key domains to consider that frequently create resource strain during COVID-19. | 2020 |
| China’s Response to the COVID-19 Outbreak: A Model for Epidemic Preparedness and Management | AlTakarli, N. S  (56) | This article presents a narrative review comparing China's response to the SARS outbreak with its response to the COVID-19 outbreak. The assessment focuses on epidemic preparedness and management strategies, including the nation's capacity for epidemic response, case detection and monitoring, healthcare infrastructure, and the readiness of medical teams, as guided by the frameworks of both the CDC and WHO. | 2020 |
| Indonesian hospital's preparedness for handling COVID-19 in the early onset of an outbreak: A qualitative study of nurse managers | Asmaningrum, N.  Ferguson, C.  Ridla, A. Z.  Kurniawati, D.  (22) | This research delves into the viewpoints of nurse managers regarding hospital readiness to tackle the early stages of the COVID-19 pandemic. Three primary categories emerged from the study: (1) operational policy, (2) infrastructure arrangement, and (3) healthcare personnel management. | 2022 |
| Hospital surge capacity in a tertiary emergency referral centre during the COVID‐19 outbreak in Italy | Carenzo, L  Costantini, E  Greco, M  Barra, FL  Rendiniello, V  Mainetti, M  et al  (57) | Describe a specialized task force to outline a response strategy encompassing: (1) the creation of specialized intensive care units specifically for patients with COVID-19; (2) the development of procedures for preliminary triage, diagnosis, and the isolation of potential and verified cases; and (3) comprehensive training for all personnel to function effectively, including the proper use of personal protective equipment and patient care. | 2020 |
| Health workforce strategies in response to major health events: a rapid scoping review with lessons learned for the response to the COVID-19 pandemic | Coates, Alison  Fuad, Asli-Oubah  Hodgson, Amanda  Bourgeault, Ivy Lynn  (25) | A scoping review examined the health workforce's reactions to infectious disease outbreaks. The described workforce approaches primarily aimed to boost the number of healthcare professionals in certain regions, enhance the adaptability of the workforce to address requirements in innovative manners, or provide backing and continuation for health workers in their roles. | 2021 |
| Strategic human resource management and COVID‐19: Emerging challenges and research opportunities | Collings, David G  McMackin, John  Nyberg, Anthony J  Wright, Patrick M  (36) | Consider the impact of COVID-19 on HR research, pinpointing essential research topics for strategic human resource management (HRM). Emphasize the pivotal function HR assumes in steering both operational and strategic triumph amid the COVID-19 crisis. | 2021 |
| Comprehensive Hospital Preparedness Checklist for Coronavirus Disease 2019 (COVID-19) 2022, CDC | (58) | The CDC offers various strategies in a checklist format for health systems and hospitals to manage the challenges posed by the COVID-19 pandemic. | 2020 |
| Rural Healthcare Center Preparation and Readiness Response to Threat of COVID-19 | Brown, J.  Guru, S.  Williams, K.  Florentino, R.  Miner, J.  Cagir, B.  (59) | The author delves into the challenges and strategies implemented within the framework of rural health networks in the USA. | 2020 |
| Secondary care: document of preparedness for COVID-19 | (60) | The article introduces a framework to bolster hospital management during the COVID-19 pandemic crisis. The components of this framework include Workforce, Infrastructure, Service Reconfiguration, Equipment, Data and Information Management, Internal Communication, External Communication, and Escalation Procedures. | 2020 |
| Structural capacity, technological human resources, and mechanical ventilation requirements in 58 intensive care units in Argentina during the SARS-CoV-2 pandemic. A SATICOVID-19 Study. | Estenssoro, Elisa  Plotnikow, Gustavo  Loudet, Cecilia I  Ríos, Fernando G  Kanoore Edul, Vanina S  Andrian, Macarena  Romero, Ignacio  Et al.  (61) | The article highlights crucial elements essential for enhancing critical operational sectors, following the augmentation of bed capacity, staff numbers, and the sufficient provision of ventilators and necessary supplies. | 2020 |
| Use of incident command system for disaster preparedness: a model for an emergency department COVID-19 response | Farcas, Andra  Ko, Justine  Chan, Jennifer  Malik, Sanjeev  Nono, Lisa  Chiampas, George  (62) | The article details how an emergency department (ED) tailored Forward Command structure aligns with the broader hospital Incident Command System (ICS). It also delves into the unique challenges posed within the ED context. Integral to this ICS are a hospital-wide incident command, often referred to as the Joint Operations Center (JOC), and the specific ED Forward Command. | 2021 |
| How can health systems better prepare for the next pandemic? Lessons learned from the management of COVID-19 in Quebec (Canada) | Alami, Hassane  Lehoux, Pascale  Fleet, Richard  Fortin, Jean-Paul  Liu, Joanne  Attieh, Randa  Et al  (13) | Utilizing the framework proposed by Palagyi et al., this article reflects upon field observations and data from the first wave of the pandemic in Quebec, Canada. The article evaluates and deliberates on the success and failures evident in Quebec's handling of COVID-19 during this period. | 2021 |
| Responding to COVID‐19: how to navigate a public health emergency legally and ethically | Gostin, Lawrence O  Friedman, Eric A  Wetter, Sarah A  (63) | The article provides ethical considerations and strategies to be mindful of during public health emergencies, such as the COVID-19 pandemic. | 2020 |
| Hospital preparedness for COVID-19 pandemic: experience from department of medicine at Veterans Affairs Connecticut Healthcare System | Gupta, Shaili  Federman, Daniel  (26) | The article details the strategies employed by the Veterans Affairs Connecticut Healthcare System in the USA amidst the COVID-19 pandemic, specifically focusing on leadership and governance, supply chain management, and human resources. | 2020 |
| Hospital Preparedness for COVID-19: The Known and The Known Unknown | Kaito, Daiki  Matsumura, Kazuki  Yamamoto, Ryo  (64) | The article presents a thoughtful analysis on "Stuff, staff, space, and governance" in shaping optimal responses during the COVID-19 crisis. | 2021 |
| Current infection prevention and control strategies of COVID-19 in hospitals | Kalin Ünüvar, G.  Doğanay, M.  Alp, E.  (40) | This article delves into the significance of strategies such as screening, personal protection measures, triage processes, and psychological support initiatives for healthcare workers, emphasizing their potential effectiveness in crisis management | 2021 |
| The Preparedness of Hospital Emergency Departments for Responding to Disasters in Iran; a Systematic Review and Meta-Analysis | Kazemzadeh, M.  Shafiei, E.  Jahangiri, K.  Yousefi, K.  Sahebi, A  (34) | This study aimed to assess the readiness of hospital emergency departments in Iran for disaster responses through a systematic review and meta-analysis. The average readiness of hospital emergency departments in addressing disasters in Iran was determined to be 54.64% (95% CI = 41.15-68.13, I2 = 0.0%; p = 0.727). | 2019 |
| Healthcare Resource Management and Pandemic Preparedness for COVID-19: A Single Centre Experience From Jodhpur, India | Khichar, S.  Midha, N.  Bohra, G. K.  Kumar, D.  Gopalakrishanan, M.  Kumar, B.  Et al  (65) | The article outlines the different facets of pandemic readiness implemented at an tertiary-care hospital in Rajasthan, India. Key areas of focus for preparedness included (a) Management of healthcare spaces, (b) Management of healthcare personnel, (c) Training for healthcare workers, and (d) Infection prevention measures. | 2020 |
| Building global health research capacity to address research imperatives following the COVID-19 pandemic | Kilmarx, Peter H  Glass, Roger I  (42) | The article underscores the significance of research and development of innovative tools and interventions to enhance health systems' ability to tackle pandemics like COVID-19, with a particular emphasis on strengthening health research capacity. | 2021 |
| Strategical preparedness and response actions in the healthcare system against coronavirus disease 2019 according to transmission scenario in Korea | Kim, Tark  Choi, Min Joo  Kim, Sun Bean  Kim, Jin Yong  Lee, Jacob  Oh, Hong Sang  (15) | The article highlights the measures of preparedness and response within the healthcare system against COVID-19, drawing on the experiences from different transmission scenarios in Korea. | 2020 |
| A national healthcare response to intensive care bed requirements during the COVID-19 outbreak in France | Lefrant, Jean-Yves  Fischer, Marc-Olivier  Potier, Hugo  Degryse, Cécile  Jaber, Samir  Muller, Lauren  Et al  (16) | The article outlines the methods by which the French Health Care system increased bed capacity amid the COVID-19 pandemic. The synergy between intensivists, critical care anaesthesiologists, emergency physicians, and the proactive involvement of nursing staff played a pivotal role in this scenario. | 2020 |
| ICU bed capacity during COVID-19 pandemic in France: From ephemeral beds to continuous and permanent adaptation | Lefrant, J. Y.  Pirracchio, R.  Benhamou, D.  Dureuil, B.  Pottecher, J.  Samain, E.  Et al  (66) | The article delves into the strategies employed by the French Health Care system to augment ICU capacity amidst the COVID-19 pandemic and the necessary adaptations undertaken to meet the challenges. | 2021 |
| Developing public health emergency response leaders in incident management: a scoping review of educational interventions | Li, Yang  Hsu, Edbert B  Pham, NhuNgoc  Davis, Xiaohong Mao  Podgornik, Michelle N  Trigoso, Silvia M  (67) | By a scoping review, the articles describe existing methods of incident management training and exercises. Five themes emerged from this work. 1) experiential learning as an established approach to foster engaging and interactive learning environments and optimize training design; (2) technology-aided decision support tools are increasingly common for crisis decision-making; (3) integration of leadership training in the education continuum is needed for developing public health response leaders; (4) equal emphasis on competency and character is needed for developing capable and adaptable leaders; and (5) consistent evaluation methodologies and metrics are needed to assess the effectiveness of educational interventions. | 2021 |
| Innovation and knowledge sharing can transform COVID-19 infection prevention response | Nathavitharana, Ruvandhi R  Patel, Payal K  Tierney, Dylan B  Mehrotra, Preeti  Lederer, Philip A  Davis, Sheila  Et al  (68) | The article details strategies to reduce infections among healthcare workers (HCWs) and emphasizes the significance of Personal Protective Equipment (PPE). | 2020 |
| Safe hospital preparedness in the era of COVID-19: The Swiss cheese model | Noh, Ji Yun  Song, Joon Young  Yoon, Jin Gu  Seong, Hye  Cheong, Hee Jin  Kim, Woo Joo  (35) | The article highlights a variety of strategies implemented by hospitals and government levels in South Korea during the COVID-19 crisis. | 2020  (35) |
| A national survey of hospital readiness during the COVID-19 pandemic in Nigeria | Ogoina, Dimie  Mahmood, Dalhat  Oyeyemi, Abisoye Sunday  Okoye, Ogochukwu Chinedum  Kwaghe, Vivian  Habib, Zayai  Et al  (17) | Utilizing a checklist adapted from WHO for hospital preparedness, the author revealed that the readiness scores spanned from 28.2% to 88.7% (with a median of 68.4%). Notably, only three hospitals, representing 15% of the total, achieved adequate readiness across all six geopolitical zones of Nigeria. | 2021 |
| Health security capacities in the context of COVID-19 outbreak: an analysis of International Health Regulations annual report data from 182 countries | Kandel, N.  Chungong, S.  Omaar, A.  Xing, J  (7) | Out of 182 nations, 52 (or 28%) possessed prevention capacities at levels 1 or 2, while 60 (or 33%) had response capacities at these levels. On the higher end, 81 countries (equivalent to 45%) demonstrated prevention capacities and another 78 countries (or 43%) showcased response capacities at levels 4 or 5, suggesting that these nations were operationally prepared. | 2020 |
| Health system preparedness for emerging infectious diseases: a synthesis of the literature | Palagyi, Anna  Marais, Ben J  Abimbola, Seye  Topp, Stephanie M  McBryde, Emma S  Negin, Joel  (33) | The article introduces a conceptual framework that identifies six fundamental constructs. four focused on material resources and structures (i.e., system 'hardware'), including (i) Surveillance, (ii) Infrastructure and medical supplies, (iii) Workforce, and (iv) Communication mechanisms; and two focused on human and institutional relationships, values and norms (i.e. system 'software'), including (i) Governance, and (ii) Trust. | 2019 |
| Flexibility during the COVID-19 Pandemic Response: Healthcare Facility Assessment Tools for Resilient Evaluation | Brambilla, Andrea  Sun, Tian-zhi  Elshazly, Waleed  Ghazy, Ahmed  Barach, Paul  FLindahl, Göran et al  (69) | Using a comprehensive five-step research methodology encompassing a literature review, examination of design guidelines, evaluation through case studies, enhancement of the assessment tool, and a final test of the revised tool, the article highlights the introduction of the Optimized Flexibility Assessment Tool (OFAT). This tool is devised to evaluate the adaptability of healthcare facilities during health emergencies. | 2021 |
| Challenges and issues about organizing a hospital to respond to the COVID-19 outbreak: experience from a French reference centre | Peiffer-Smadja, Nathan  Lucet, J-C  Bendjelloul, Gisèle  Bouadma, Lila  Gerard, Sandrine  Choquet, Christophe  (70) | The article details the essential components of the response to COVID-19 at the hospital level, drawing insights from the experiences faced by Bichat-Claude Bernard hospital in Paris, including the challenges encountered and potential issues to be mindful of in the future. | 2020 |
| Ethical dilemmas due to the Covid-19 pandemic | Robert, René  Kentish-Barnes, Nancy  Boyer, Alexandre  Laurent, Alexandra  Azoulay, Elie  Reignier, Jean  (44) | The article highlights several pivotal ethical decisions that ICU caregivers grappled with during the COVID-19 pandemic and emphasizes their inherent limitations. | 2020 |
| Applying Syndemic Theory to Acute Illness | Rudd, Kristina E.  Mair, Christina F.  Angus, Derek C  (71) | The article emphasizes the application of Syndemic Theory to complex systems during public health crises. | 2022 |
| Disability, ethics, and health care in the COVID-19 pandemic | (45)  Sabatello, Maya  Burke, Teresa Blankmeyer  McDonald, Katherine E  Appelbaum, Paul S | The article underscores that reactions to the pandemic need to adhere to legal standards, uphold principles of distributive justice, and respect societal values of safeguarding vulnerable groups - fundamental tenets of public health. | 2020 |
| The Lancet Commission on lessons for the future from the COVID-19 pandemic | Sachs, Jeffrey D  Karim, Salim S Abdool  Aknin, Lara  Allen, Joseph  Brosbøl, Kirsten  Colombo, Francesca  (1) | The author pinpointed 10 primary observations regarding health system responsiveness globally and subsequently provided 11 essential recommendations. | 2022 |
| Developing a hospital preparedness checklist to assess the ability to respond to the COVID-19 pandemic | Seyedin, Hesam  Moslehi, Shandiz  Sakhaei, Fazeleh  Dowlati, Mohsen  (37) | The article presents a checklist designed to assess the readiness of hospitals in addressing the challenges posed by the COVID-19 pandemic. the final checklist had 2 main domains: measures at national and measures at hospital level. Preparedness at national level was categorized into 3 aspects that are implemented by the health ministry. Preparedness at hospital level was categorized in 24 subgroups. | 2021 |
| Preparedness for coronavirus disease in hospitals of Nepal: a nationwide survey | Shrestha, Gentle Sunder  Paneru, Hem Raj  Acharya, Subhash Prasad  Shrestha, Sanjeet Krishna  Sigdel, Mahesh Raj  Tiwari, Sanjeeb  (72) | That most of the hospitals in Nepal are not well prepared for management of patients with COVID-19. Resource allocation and policy making should be aimed to enhance national preparedness for the pandemic. | 2020 |
| Addressing challenges for clinical research responses to emerging epidemics and pandemics: a scoping review | Sigfrid, Louise  Maskell, Katherine  Bannister, Peter G  Ismail, Sharif A  Collinson, Shelui  Regmi, Sadie  (43) | For a robust global readiness and action plan against COVID-19 and potential future outbreaks, it's imperative to execute identified strategies for swift clinical research rollout, application, and distribution. Immediate enhancements in collaborations, funding structures, international and domestic research competencies, especially in regions susceptible to widespread diseases, are essential. | 2020 |
| Perceived challenges of COVID-19 infection prevention and control preparedness: A multinational survey | Tartari, E.  Hopman, J.  Allegranzi, B.  Gao, B.  Widmer, A.  Cheng, V. C.  (73) | The authors assessed the perceptions of infection preventionists on the current global IPC preparedness measures for COVID-19. Of all participants, 66.6% were aware of the existence of national guidelines to prevent COVID-19. A shortage of [personal protective equipment](https://www.sciencedirect.com/topics/medicine-and-dentistry/personal-protective-equipment) (PPE) supplies was reported by 48% | 2020 |
| The COVID-19 pandemic and healthcare systems in Africa: a scoping review of preparedness, impact and response | Tessema, Gizachew A  Kinfu, Yohannes  Dachew, Berihun Assefa  Tesema, Azeb Gebresilassie  Assefa, Yibeltal  Alene, Kefyalew Addis  Et al  (4) | Primary challenges in health system readiness were the scarcity of necessary health services for the pandemic, insufficient resources and equipment, and restricted testing capabilities and surge capacity for COVID-19. A decline in patient visits and missed scheduled appointments stood out as prevalent consequences of the COVID-19 pandemic. Identified health system reactions in the review encompassed introducing telephone consultations, repurposing existing services, establishing isolation centers, and offering COVID-19 guidelines in certain contexts. | 2021 |
| Hospital Preparedness and Response Framework during infection pandemic | Thapa, Bikash Bikram  (2) | The strategic domain for hospital preparedness for COVID-19 pandemic related mass infection incident were: 1. Leadership 2. Ethics 3. Clinical care 4. Resources 5. Infection prevention and control 6. Data management, which were bonded together in action by effective communication, training and education, and research | 2021 |
| Knowledge management based on information technology in response to COVID-19 crisis | Wang, Wei-Tsong  Wu, Su-Ying  (74) | This research delves into the utilization of pioneering IT-driven solutions to minimize exposure risks, emphasizing the role of an AI-driven epidemic intelligence dashboard. By using the functioning of healthcare organizations in Taiwan during the COVID-19 crisis as a reference, the study showcases the efficiency of integrating IT-enabled Knowledge Management practices within Crisis Management frameworks. Such integrations prove instrumental in preventing or mitigating negative outcomes of crises. | 2021 |
| Covid-19 pandemic: A frontline hospital reorganization to cope with therapeutic and diagnostic emergency | De Filippis, G.  Cavazzana, L.  Gimigliano, A.  Piacenza, M.  Vimercati, S.  Et al  (75) | The article emphasizes the importance of crafting an emergency plan during non-crisis periods to address extraordinary events like a pandemic. Such a plan should include ongoing training, regular revisions, and encompass all departments of the hospital structure. | 2020 |
| Hospital preparedness for mass critical care during SARS-CoV-2 pandemic | Wurmb, Thomas  Scholtes, Katja  Kolibay, Felix  Schorscher, Nora  Ertl, Georg  Ernestus, Ralf-Ingo  (76) | This article provides an overview of the preparedness and action steps advised for acute care hospitals. These recommendations encompass leadership approaches, communication of healthcare infection control decisions, resource and supply management, strategies for staff and space to prevent in-hospital transmissions, and handling of elective patient care. | 2020 |
| Lessons learnt from the COVID-19 pandemic: results of EAHP survey on the future crisis preparedness of hospital pharmacies | Leonardi Vinci, D.  Polidori, P.  Miljković, N.  Batista, A.  Amann, S.  Makridaki, D.  Et al.  (77) | The article centers on the scarcity of medical supplies and delves into the experiences of hospitals during the initial stage of the crisis. | 2022 |
| Main Factors Affecting the Readiness and Responsiveness of Healthcare Systems during Epidemic Crises: A Scoping Review on Cases of SARS, MERS, and COVID-19 | Mohammadpour, M.  Zarifinezhad, E.  Ghanbarzadegan, A.  Naderimanesh, K.  Shaarbafchizadeh, N.  Bastani, P | During the outbreaks of severe acute respiratory syndrome (SARS), Middle East respiratory syndrome (MERS), and COVID-19, five primary elements influenced the readiness and response of nations: Community-related interventions, Managerial interventions, Socioeconomic factors, Preparedness of hospitals and health centers, and Environmental factors. Each of these primary themes is further divided, resulting in 38 associated sub-themes. | 2021 |

**REFERENCES**

1. Sachs JD, Karim SSA, Aknin L, Allen J, Brosbøl K, Colombo F, et al. The Lancet Commission on lessons for the future from the COVID-19 pandemic. The Lancet. 2022;400(10359):1224-80.

2. Thapa BB. Hospital Preparedness and Response Framework during infection pandemic. 2021:2021.06.28.21259630.

3. Bell L, van Gemert C, Merilles OE, Cash HL, Stoové M, Hellard M. The impact of COVID-19 on public health systems in the Pacific Island Countries and Territories. The Lancet Regional Health–Western Pacific 2022;25.

4. Tessema GA, Kinfu Y, Dachew BA, Tesema AG, Assefa Y, Alene KA, et al. The COVID-19 pandemic and healthcare systems in Africa: a scoping review of preparedness, impact and response. BMJ global health 2021;6(12):e007179.

5. Horton R. Offline: COVID-19 is not a pandemic. The lancet. 2020;396(10255):874.

6. Chang AY, Cullen MR, Harrington RA, Barry MJJoIM. The impact of novel coronavirus COVID‐19 on noncommunicable disease patients and health systems: a review. 2021;289(4):450-62.

7. Kandel N, Chungong S, Omaar A, Xing J. Health security capacities in the context of COVID-19 outbreak: an analysis of International Health Regulations annual report data from 182 countries. Lancet. 2020;395(10229):1047-53.

8. Kontopantelis E, Mamas MA, Deanfield J, Asaria M, Doran T. Excess mortality in England and Wales during the first wave of the COVID-19 pandemic. Epidemiol Community Health. 2021;75(3):213-23.

9. Wei C, Lee C, Hsu T, Hsu W, Chan C, Chen S, et al. Correlation of population mortality of COVID-19 and testing coverage: a comparison among 36 OECD countries. J Epidemiology

Infection 2021;149:e1.

10. Best LA, Law MA, Roach S, Wilbiks J. The psychological impact of COVID-19 in Canada: Effects of social isolation during the initial response. Canadian Psychology/Psychologie Canadienne 2021;62(1):143.

11. Sutton J, Arku G. The importance of local characteristics: An examination of Canadian cities' resilience during the 2020 economic crisis. The Canadian Geographer/Le Géographe canadien 2022;66(4):712-27.

12. Anesi GL, Lynch Y, Evans L. A Conceptual and Adaptable Approach to Hospital Preparedness for Acute Surge Events Due to Emerging Infectious Diseases. Crit Care Explor. 2020;2(4):e0110-e.

13. Alami H, Lehoux P, Fleet R, Fortin J-P, Liu J, Attieh R, et al. How can health systems better prepare for the next pandemic? Lessons learned from the management of COVID-19 in Quebec (Canada). Frontiers in public health. 2021;9:671833.

14. Cunningham JM, Persoff J, Piper C, Burger A, Shinnar E, Cunnius P, et al. A Framework for Hospital Medicine's Involvement in Disaster Preparedness and Response. Health security

2022;20(2):172-6.

15. Kim T, Choi MJ, Kim SB, Kim JY, Lee J, Oh HS, et al. Strategical preparedness and response actions in the healthcare system against coronavirus disease 2019 according to transmission scenario in Korea. Infection chemotherapy 2020;52(3):389.

16. Lefrant J-Y, Fischer M-O, Potier H, Degryse C, Jaber S, Muller L, et al. A national healthcare response to intensive care bed requirements during the COVID-19 outbreak in France. Anaesthesia Critical Care & Pain Medicine. 2020;39(6):709-15.

17. Ogoina D, Mahmood D, Oyeyemi AS, Okoye OC, Kwaghe V, Habib Z, et al. A national survey of hospital readiness during the COVID-19 pandemic in Nigeria. PLOS ONE. 2021;16(9):e0257567.

18. Governement. Provincial Pandemic Coordination Plan. Governement of New Brunswick 2020 2020. Report No.: 978-1-4605-2458-9.

19. Hollnagel E. Safety-II in practice: developing the resilience potentials: Taylor & Francis; 2017.

20. Patriarca R, Di Gravio G, Costantino F, Falegnami A, Bilotta F. An Analytic Framework to Assess Organizational Resilience. Saf Health Work. 2018;9(3):265-76.

21. Adelaja I, Sayma M, Walton H, McLachlan G, de Boisanger J, Bartlett-Pestell S, et al. A comprehensive hospital agile preparedness (CHAPs) tool for pandemic preparedness, based on the COVID-19 experience. Future Healthcare Journal. 2020;7(2):165.

22. Asmaningrum N, Ferguson C, Ridla AZ, Kurniawati D. Indonesian hospital's preparedness for handling COVID-19 in the early onset of an outbreak: A qualitative study of nurse managers. Australas Emerg Care. 2022.

23. Augustin M, Schommers P, Suárez I, Koehler P, Gruell H, Klein F, et al. Rapid response infrastructure for pandemic preparedness in a tertiary care hospital: lessons learned from the COVID-19 outbreak in Cologne, Germany, February to March 2020. Eurosurveillance. 2020;25(21):2000531.

24. Beyramijam M, Khankeh H, Shahabi-Rabori MA, Aminizadeh M, Sheikhbardsiri H. Hospital disaster preparedness in Iranian province: A cross-sectional study using a standard tool. Am J Disaster Med. 2021;16(3):233-9.

25. Coates A, Fuad A-O, Hodgson A, Bourgeault IL. Health workforce strategies in response to major health events: a rapid scoping review with lessons learned for the response to the COVID-19 pandemic. Human Resources for Health. 2021;19(1):154.

26. Gupta S, Federman D. Hospital preparedness for COVID-19 pandemic: experience from department of medicine at Veterans Affairs Connecticut Healthcare System. Postgraduate Medicine. 2020;132(6):489-94.

27. Coles E, Wells M, Maxwell M, Harris FM, Anderson J, Gray NM, et al. The influence of contextual factors on healthcare quality improvement initiatives: what works, for whom and in what setting? Protocol for a realist review. Systematic Reviews. 2017;6(1):168.

28. Schloemer T, Schröder-Bäck P. Criteria for evaluating transferability of health interventions: a systematic review and thematic synthesis. Implementation Science. 2018;13(1):88.

29. Damschroder LJ, Aron DC, Keith RE, Kirsh SR, Alexander JA, Lowery JC. Fostering implementation of health services research findings into practice: a consolidated framework for advancing implementation science. Implementation Science. 2009;4(1):50.

30. Föhn Z, Nicolet A, Marti J, Kaufmann C, Balthasar A. Stakeholder engagement in designing attributes for a discrete choice experiment with policy implications: an example of two Swiss studies on healthcare delivery. Value in Health. 2023.

31. Chaves BG, Briand C, Bouabida K. Innovation in healthcare organizations: Concepts and challenges to consider. International Journal of Health Research and Innovation. 2021;9(1).

32. World Health Organization. Hospital emergency response checklist: an all-hazards tool for hospital administrators and emergency managers. World Health Organization. Regional Office for Europe; 2011.

33. Palagyi A, Marais BJ, Abimbola S, Topp SM, McBryde ES, Negin J. Health system preparedness for emerging infectious diseases: a synthesis of the literature. Global Public Health. 2019;14(12):1847-68.

34. Kazemzadeh M, Shafiei E, Jahangiri K, Yousefi K, Sahebi A. The Preparedness of Hospital Emergency Departments for Responding to Disasters in Iran; a Systematic Review and Meta-Analysis. Arch Acad Emerg Med. 2019;7(1):e58.

35. Noh JY, Song JY, Yoon JG, Seong H, Cheong HJ, Kim WJ. Safe hospital preparedness in the era of COVID-19: The Swiss cheese model. International journal of infectious diseases. 2020;98:294-6.

36. Collings DG, McMackin J, Nyberg AJ, Wright PM. Strategic human resource management and COVID‐19: Emerging challenges and research opportunities. Journal of Management Studies

2021.

37. Seyedin H, Moslehi S, Sakhaei F, Dowlati M. Developing a hospital preparedness checklist to assess the ability to respond to the COVID-19 pandemic. East Mediterr Health 2021;2:131-41.

38. West M, Armit K, Loewenthal L, Eckert R, West T, Lee A. Leadership and leadership development in health care: the evidence base. 2015.

39. Beilstein CM, Lehmann LE, Braun M, Urman RD, Luedi MM, Stüber F. Leadership in a time of crisis: Lessons learned from a pandemic. Best Practice & Research Clinical Anaesthesiology. 2021;35(3):405-14.

40. Kalin Ünüvar G, Doğanay M, Alp E. Current infection prevention and control strategies of COVID-19 in hospitals. Turk J Med Sci. 2021;51(Si-1):3215-20.

41. Cattelan AM, Sasset L, Di Meco E, Cocchio S, Barbaro F, Cavinato S, et al. An Integrated Strategy for the Prevention of SARS-CoV-2 Infection in Healthcare Workers: A Prospective Observational Study. Int J Environ Res Public Health. 2020;17(16).

42. Kilmarx PH, Glass RI. Building global health research capacity to address research imperatives following the COVID-19 pandemic. PLoS Medicine. 2021;18(8):e1003753.

43. Sigfrid L, Maskell K, Bannister PG, Ismail SA, Collinson S, Regmi S, et al. Addressing challenges for clinical research responses to emerging epidemics and pandemics: a scoping review. BMC medicine. 2020;18(1):1-15.

44. Robert R, Kentish-Barnes N, Boyer A, Laurent A, Azoulay E, Reignier J. Ethical dilemmas due to the Covid-19 pandemic. Annals of intensive care. 2020;10(1):1-9.

45. Sabatello M, Burke TB, McDonald KE, Appelbaum PS. Disability, ethics, and health care in the COVID-19 pandemic. American Journal of Public Health

2020;110(10):1523-7.

46. Collings DG, McMackin J, Nyberg AJ, Wright PM. Strategic human resource management and COVID‐19: Emerging challenges and research opportunities. Journal of Management Studies

2021.

47. Khan Y, O’Sullivan T, Brown A, Tracey S, Gibson J, Généreux M, et al. Public health emergency preparedness: a framework to promote resilience. BMC public health. 2018;18(1):1-16.

48. Koka PM, Sawe HR, Mbaya KR, Kilindimo SS, Mfinanga JA, Mwafongo VG, et al. Disaster preparedness and response capacity of regional hospitals in Tanzania: a descriptive cross-sectional study. BMC Health Serv Res. 2018;18(1):835.

49. Adams L. Exploring the concept of surge capacity. The Online Journal of Issues in Nursing

2009;14(2).

50. Kelen GD, McCarthy ML. The science of surge. Academic Emergency Medicine 2006;13(11):1089-94.

51. Hasan MK, Nasrullah SM, Quattrocchi A, Arcos González P, Castro Delgado R. Hospital Surge Capacity Preparedness in Disasters and Emergencies: Protocol for a Systematic Review. Int J Environ Res Public Health. 2022;19(20).

52. World Health Organization. Hospital readiness checklist for COVID-19 interim version February 24 2020. World Health Organization. Regional Office for Europe; 2020.

53. Centers for Disease Control Prevention. Coronavirus disease 2019 (COVID-19) hospital preparedness assessment tool. 2020.

54. Frawley T, van Gelderen F, Somanadhan S, Coveney K, Phelan A, Lynam-Loane P, et al. The impact of COVID-19 on health systems, mental health and the potential for nursing. Irish Journal of Psychological Medicine. 2021;38(3):220-6.

55. Hacker KA, Briss PA, Richardson L, Wright J, Petersen R. Peer reviewed: COVID-19 and chronic disease: the impact now and in the future. J Preventing Chronic Disease. 2021;18.

56. AlTakarli NS. China’s Response to the COVID-19 Outbreak: A Model for Epidemic Preparedness and Management. Dubai Medical Journal. 2020;3(2):44-9.

57. Carenzo L, Costantini E, Greco M, Barra F, Rendiniello V, Mainetti M, et al. Hospital surge capacity in a tertiary emergency referral centre during the COVID‐19 outbreak in Italy. Anaesthesia Critical Care & Pain Medicine. 2020;75(7):928-34.

58. Centers for Disease Control Prevention. Comprehensive hospital preparedness checklist for coronavirus disease 2019 (COVID-19). US Department of Health Human Services.; 2020.

59. Brown J, Guru S, Williams K, Florentino R, Miner J, Cagir B. Rural Healthcare Center Preparation and Readiness Response to Threat of COVID-19. J Am Coll Surg. 2020;230(6):1105-10.

60. National Health Service (NHS). Secondary care: document of preparedness for COVID-19. England; 2020.

61. Estenssoro E, Plotnikow G, Loudet CI, Ríos FG, Kanoore Edul VS, Andrian M, et al. Capacidad estructural, recursos humanos tecnológicos y requerimientos de ventilación mecánica en 58 unidades de cuidados intensivos en argentina durante la pandemia por SARS-CoV-2. Estudio SATICOVID-19. Medicina. 2022;82(1):35-46.

62. Farcas A, Ko J, Chan J, Malik S, Nono L, Chiampas GJDm, et al. Use of incident command system for disaster preparedness: a model for an emergency department COVID-19 response. 2021;15(3):e31-e6.

63. Gostin LO, Friedman EA, Wetter SA. Responding to COVID‐19: how to navigate a public health emergency legally and ethically. Hastings center report. 2020;50(2):8-12.

64. Kaito D, Matsumura K, Yamamoto R. Hospital Preparedness for COVID-19: The Known and The Known Unknown. The Keio Journal of Medicine. 2021;70(2):25-34.

65. Khichar S, Midha N, Bohra GK, Kumar D, Gopalakrishanan M, Kumar B, et al. Healthcare Resource Management and Pandemic Preparedness for COVID-19: A Single Centre Experience From Jodhpur, India. Int J Health Policy Manag. 2020;9(11):493-5.

66. Lefrant JY, Pirracchio R, Benhamou D, Dureuil B, Pottecher J, Samain E, et al. ICU bed capacity during COVID-19 pandemic in France: From ephemeral beds to continuous and permanent adaptation. Anaesth Crit Care Pain Med. 2021;40(3):100873.

67. Li Y, Hsu EB, Pham N, Davis XM, Podgornik MN, Trigoso SM. Developing public health emergency response leaders in incident management: a scoping review of educational interventions. Disaster medicine public health preparedness 2021:1-30.

68. Nathavitharana RR, Patel PK, Tierney DB, Mehrotra P, Lederer PA, Davis S, et al. Innovation and knowledge sharing can transform COVID-19 infection prevention response. Journal of Hospital Medicine. 2020;15(5):299.

69. Brambilla A, Sun T-z, Elshazly W, Ghazy A, Barach P, Lindahl G, et al. Flexibility during the COVID-19 Pandemic Response: Healthcare Facility Assessment Tools for Resilient Evaluation. 2021;18(21):11478.

70. Peiffer-Smadja N, Lucet J-C, Bendjelloul G, Bouadma L, Gerard S, Choquet C, et al. Challenges and issues about organizing a hospital to respond to the COVID-19 outbreak: experience from a French reference centre. Clinical Microbiology. 2020;26(6):669-72.

71. Rudd KE, Mair CF, Angus DC. Applying Syndemic Theory to Acute Illness. JAMA. 2022;327(1):33-4.

72. Shrestha GS, Paneru HR, Acharya SP, Shrestha SK, Sigdel MR, Tiwari S, et al. Preparedness for coronavirus disease in hospitals of Nepal: a nationwide survey. Journal of the Nepal Medical Association. 2020;58(224):248.

73. Tartari E, Hopman J, Allegranzi B, Gao B, Widmer A, Cheng VC, et al. Perceived challenges of COVID-19 infection prevention and control preparedness: A multinational survey. J Glob Antimicrob Resist. 2020;22:779-81.

74. Wang W-T, Wu S-Y. Knowledge management based on information technology in response to COVID-19 crisis. Knowledge management research 2021;19(4):468-74.

75. De Filippis G, Cavazzana L, Gimigliano A, Piacenza M, Vimercati S. Covid-19 pandemic: A frontline hospital reorganization to cope with therapeutic and diagnostic emergency. Pharmacol Res. 2020;161:105160.

76. Wurmb T, Scholtes K, Kolibay F, Schorscher N, Ertl G, Ernestus R-I, et al. Hospital preparedness for mass critical care during SARS-CoV-2 pandemic. Critical Care. 2020;24(1):386.

77. Leonardi Vinci D, Polidori P, Miljković N, Batista A, Amann S, Makridaki D, et al. Lessons learnt from the COVID-19 pandemic: results of EAHP survey on the future crisis preparedness of hospital pharmacies. Eur J Hosp Pharm. 2022;29(5):242-7.
