# Supplementary material for: Assessing healthcare capacity crisis preparedness: development of an evaluation tool by a Canadian health authority
Source: Front Public Health. 2023 Oct 10;11:1231738. doi: 10.3389/fpubh.2023.1231738 (PMC10594116; doi:10.3389/fpubh.2023.1231738)
Supplement: Supplementary file 3 [file Data_Sheet_1.PDF]

Research and Health Evaluation Unit

HEALTH CRISIS MANAGEMENT CAPACITY
Evaluation Tool

RÉSEAU DE SANTÉ vitalité HEALTH NETWORK

Use of this evaluation tool

1. This tool encompasses 8 dimensions, 74 strategies and 109 observable items.
2. It is recommended that the evaluation of each dimension be conducted by an interdisciplinary panel.
3. The observable items are references for the evaluators; however, other items may be taken into consideration by the panel tasked with conducting the evaluation.
4. The score must be attributed based on interviews with key actors for each dimension, document analysis and observations on the ground.
5. Observations must be included to justify the score and facilitate the development of recommendations.

Overview of weighting

Total number of strategies: 74

Total number of observable items: 109

Weighting of conceptual framework dimensions

Dimension	Proposed weighting
A. Clinical Care Management	20%
B. Resources	20%
C. Governance and Leadership	10%
D. Communication and Technologies	10%
E. Infection Prevention and Control	20%
F. Health Research	5%
G. Ethics and Values	5%
H. Training	10%
Total	100%

A. Clinical Care Management

- **Weighting: 20%**
  - Number of strategies: 11
  - Observable items: 21
- **Total score: 22**
  - Variation: 0-100%

STRATEGIES	OBSERVABLE ITEMS	POINTS	OBSERVATIONS	FINAL SCORE
S1.1 Available, accessible and accredited laboratories	1.1 Accreditation by an organization **(1)**			
	1.2 Average time for PCR – COVID-19<br>• Less than 24h **(1)**<br>OR<br>• Between 24-48h **(0.5)**			
S.1.2 Laboratory information management system that allows rapid integration of data and strategic information	1.3 Integrated computer system of laboratories from all regions **(1)**			
	1.4 Potential integration of patient records **(0.5)**			
	1.5 Ease of data extraction or business intelligence (BI) tools **(0.5)**			
S.1.3 Sample transportation mechanisms based on the required security level, including national level	1.6 Sample transportation plans or protocols based on security level **(1)**			

	1.7 Qualified staff to transport samples **(1)**			
**S.1.4 Clinical protocols for diagnosis, treatment and follow-up of infectious diseases based on clinical status (e.g., COVID-19)**	1.8 Clinical protocols for common infectious diseases **(1)**			
	1.9 Proof of following and monitoring strategic protocols **(1)**			
**S.1.5 Validated greeting and triage protocol for sick patients adapted for a crisis situation**	1.10 Proof of existing triage protocols **(1)**<br><br>E.g., Manchester, SOFA, START, etc.			
	1.11 Proof of implementation **(1)**			
**S.1.6 Capacity to categorize and manage clinical conditions not associated with the pandemic**	1.12 Contingency plan for managing non-urgent cases **(2)**<br><br>E.g., based on the priority level (low, high, urgent)			
**S1.7 Presence of experts to assess new therapeutic technologies (medications, equipment, etc.) related to the health crisis**	1.13 Multidisciplinary committee to assess the integration of new therapeutic technologies **(2)**			
**S 1.8 Integrated computer system for patient management (medical,**	1.14 Systems integration **(1)**			

laboratory and radiology records) to analyze trends and visualize key indicators	1.15 Scorecards of information from the integrated system **(1)**<br><br>E.g., business intelligence tools			
S 1.9 Capacity to adjust, adapt and establish clinical protocols based on clinical needs	1.16 Clinical protocol creation and management committee **(1)**			
	1.17 Proof of meetings held **(1)**			
S1.10 Accessible, available and accredited pharmacy services	1.18 Number of adequate and accessible pharmacies within the Network **(1)**			
	1.19 Accredited pharmacies **(0.5)**			
	1.20 Adequate ratio of pharmacists **(0.5)**			
S1.11 Capacity of the Network to cope with "critical" medication shortages through strategy development	1.21 Medication transfer or contingency plan fostering the management and integration of medications between the Network's hospitals **(2)**			
			TOTAL Section A	/22
			A = (Total/22) X	A =

B.

Resources

(procurement – logistics – labour force)

2. RESOURCES – PROCUREMENT

- Weighting: 20%
  - Number of strategies: 14
  - Observable items: 16
- Total score: 28
  - Variation: 0-100%

STRATEGIES	OBSERVABLE ITEMS	POINTS	OBSERVATION	FINAL SCORE
S2.1 Resource procurement plan based on "progressive scaling" to keep the supply chain and work teams operational	2.1 Contingency plan for managing resources in a crisis context (2)<br><br>E.g., conventional capacity, emergency capacity and capacity in health crisis			
S2.2 Stocks of personal protective equipment to operate for 90 days during a crisis	2.2 Stocks of PPE available for 90 days (2)			

**S2.3 Capacity to expand the emergency services structure as well as the number of clinical inpatient beds and the intensive care beds based on the magnitude of the crisis**	2.3 Contingency plan to expand the number of beds **(2)**<br><br>E.g., mobile patient beds, moving patients with chronic illnesses, etc.			
**S2.4 Capacity to manage an oxygen shortage**	2.4 Oxygen shortage management plan **(2)**			
**S2.5 Capacity to manage shortage of essential equipment (e.g., mechanical ventilators, etc.).**	2.5 Contingency plan to acquire and manage essential equipment **(2)**			
**S2.6 Automated procurement management system to ensure quick analysis of stocks**	2.6 Automated stock management system**(2)**			
**S2.7 Capacity to access provincial reserve stocks**	2.7 Integration of stock systems into the provincial system **(2)**			
**S2.8 Capacity to deploy screening clinics based on the crisis level**	2.8 Operational plan for the deployment of screening clinics **(2)**			
**S2.9 Capacity to deploy large-scale vaccination clinics**	2.9 Operational plan for the deployment of vaccination clinics (2)			

HUMAN RESOURCES COMPONENT				
**S2.10 Capacity to redeploy staff and recruit staff based on the crisis level**	S2.10 Staff redeployment and staff recruitment plan based on the crisis level **(2)**			
**S2.11 Incentives to attract and retain health care workers**	2.11 Presence of strategies to retain talents in the context of a crisis **(2)**			

S2.12 Incentives to attract and retain health care workers in rural areas	2.12 Presence of strategies to retain talents in rural areas in the context of a crisis (2)			
S2.13 Sufficient staff based on the crisis level (e.g., physicians, nurses, etc.).	2.13 Adequate physician-nurse ratio for the population receiving services (1)<br>2.14 Plan to ensure a ratio that is 30% higher than recommended in the time of a crisis (1)			
S.14 Capacity to mobilize volunteers that can perform a variety of tasks as needed	2.15. Pool of volunteers (1)			
	2.16 Mapping of volunteers' skills (1)			
			TOTAL Section B	/28
			B = (Total/28) X 100	B =

C. Governance and Leadership

- **Weighting: 10%**
  - Number of strategies: 9
  - Observable items: 16
- **Total score: 18**
  - Variation: 0-100%

STRATEGIES	OBSERVABLE ITEMS	POINTS	OBSERVATION	FINAL SCORE
S3.1 Presence of intervention command centres (general and regional) with a multidisciplinary team at many levels	3.1 Composition of the ECC (emergency coordination centre) and the EOC (emergency operations centre) (1)			
	3.2 Proof of weekly meetings (1)			
S3.2 Clinician participation in clinical decision making	3.3 Clinical representation within governance committees (1)			
	3.4 Proof of clinical participation in meetings (1)<br><br>E.g., meeting record			

S3.3 Presence of the leadership team to facilitate communication with the organization's external partners (e.g., Department of Health, private businesses, politicians, etc.) with a view to harnessing the necessary resources and raising the awareness of political officials about the organization's internal needs	3.5 Committee tasked with external negotiation regarding resources (1)			
	3.6 Proof of activities (1)<br><br>E.g., meeting record, emails, videos			
S3.4 Participation of community members in decision making (e.g., community advisory committee)	3.7 Representation of community members within governance committees (1)			
	3.8 Proof of participation in meetings (1)<br><br>E.g., meeting record			
S3.5 Coordination and effective orientation with the Department of Health and other external partners	3.9 Committee comprised of members from the Network and the Department of Health or other partners (1)			
	3.10 Proof of meetings (1)<br><br>E.g., meeting record, emails, videos			
S3.6 Effective internal coordination between the various zones, various departments and various hospitals	3.11 Weekly meeting of the leadership team / managers of each zone to discuss issues, find common solutions and share winning strategies (1)			

	3.12 Proof of meetings (e.g., record, emails, videos, etc.) **(1)**			
S3.7 Availability of an internal leadership training and professional development program	3.13 Internal leadership training program **(1)**			
S3.8 Capacity to organize online information sessions on a regular basis with the leadership team	3.14 Online information session on a weekly basis for the teams on the ground during the crisis (CEO/VP)<br><br>One per week **(2)**<br>**OR**<br>One every 15 days **(1)**<br><br>Proof of meetings (e.g., record, emails, videos, etc.)			
S3.9 Adaptive, supportive, open and caring leadership model	3.15 Measurement of the organizational climate of the zones by the Network (1)			
	3.16 Favourable organizational climate overall **(1)**			

TOTAL Section C	/18
C = (Total/18) X 100	C =

D. Communication and Technologies

- **Weighting: 10%**
  - Number of strategies: 8
  - Observable items: 14
- **Total score: 16**
  - Variation: 0-100%

STRATEGIES	OBSERVABLE ITEMS	POINTS	OBSERVATION	FINAL SCORE
S4.1 Dissemination of command centre (EOC/ECC) decision making	4.1 Strategies or plan for dissemination of EOC/ECC decision making **(1)**			
	4.2 Dissemination timeline (less than one week) **(1)**			
S4.2 Capacity for internal and external communication, including strategies for communicating risks	4.3 Internal communication plan for the crisis context **(1)**			
	4.4 External communication plan for the crisis context **(1)**			
S4.3 Spokesperson from the organization with appropriate training for communication in crisis situations	4.5 Determination of official spokespeople for the organization **(1)**			
	4.6 Spokesperson with training for communication in crisis situations **(1)**			
S4.4 Use of social media for sharing relevant information quickly	4.7 Social media management plan in the crisis context **(1)**			
	4.8 Presence on at least two social media channels **(0.5)**			
	4.9 Proof of posts for crisis **(0.5)**<br><br>E.g., see the posts			

S4.5 Capacity to manage crisis-related misinformation on social media	4.10 Social media management plan that takes into consideration strategies to overcome misinformation on the current crisis **(2)**			
S4.6 Tool that allows quick and dynamic visualization of key crisis indicators simultaneously by the leadership team and employees	4.11 Availability of a dynamic scorecard, reports, etc., that allow quick visualization of key crisis management indicators by the leadership team **(1)**			
	4.12 Dissemination of information in the organization **(1)**			
S4.7 Document centre up to date, accessible and easy to use	4.13 Common location (online) to access key documents on the crisis adapted to everyone **(2)**			
S4.8 Availability of dynamic communication platforms	4.14 Presence of communication platforms **(2)**<br><br>E.g., Go.Data, Outbreak SharePoint, COVID-19 Tracker, Teams, Website, etc.			
			TOTAL Section D	/16
			D = (Total/16) X 100	D =

E. Infection Prevention and Control

- **Weighting: 20%**
  - Number of strategies: 16
  - Observable items: 21
- **Total score: 32**
  - Variation: 0-100%

STRATEGIES	OBSERVABLE ITEMS	POINTS	OBSERVATION	FINAL SCORE
S5.1 Personal protective equipment (PPE) available based on exposure risk in the various workstations	5.1 Availability of PPE in all units / all departments for all risk levels (well-established biosafety level) **(2)**<br><br>E.g., visors, gowns, masques, N95, etc.			
S5.2 Clear and appropriate directives on the use of PPE	5.2 Presence of protocols on the use and application of PPE **(2)**			

S5.3 Printed signage models on precautions and current operating procedures	5.3 Signage models available covering the main items (2)<br><br>E.g., + cases, suspected cases, confirmed cases, protocols, etc.			
S5.4 Multimedia signage on precautions and current operating procedures	5.4 Availability of multimedia tools for disseminating operating procedures during the crisis (2)			
S5.5 Clear visual and spatial separation of care zones to prevent exposure and reduce risks	5.5 Interior layout plan of facilities/units/departments to reduce the spread of pathogens during the crisis (2)<br><br>E.g., marking of red, yellow and green zones, etc.			
S5.6 Capacity to isolate or confine individuals who are ill or suspected cases (patients and employees)	5.6 Operating plan for isolation and confinement of individuals who are ill or suspected cases (patients and employees) (1)			
	5.7 Isolation beds available or plan for rapid increase of them (1)			
S5.7 Institutional policy on infectious disease prevention and control	5.8 Organizational infectious disease prevention and control plan (1)			
	5.9 Monitoring of strategic indicators on infectious disease prevention and control (1)			
S5.8 Institutional policy on managing inpatient visits	5.10 Plan for managing patient visits during the crisis (2)			
S5.9 Capacity to monitor and support sick employees	5.11 Internal policy on monitoring and supporting sick employees (1)			

	5.12 Specific group charged with this task (1)			
S5.10 Monitoring of health professionals' percentage level of compliance with disinfection techniques	5.13 Monitoring of employee compliance with hand washing techniques (2)			
S5.11 Policy on mobilization and use of champions or ambassadors for infection prevention and control throughout the Network	5.14 Plan for identifying and training ambassadors in infection prevention and control (2)			
S5.12 Key experts assigned to local IPC department	5.15 Microbiologists (0.5)			
	5.16 Epidemiologists (0.5)			
	5.17 Public Health (1)			
S5.13 Management of corpses	5.18 Corpse management plan (2)			
S5.14 Management and circulation of clean and soiled material well defined and marked	5.19 Plan for clean and soiled material management and circulation during a health crisis (2)			
S5.15 IPC communication strategies	5.20 IPC Department communication plan for the entire organization (2)			

S5.16 Staff trained to manage a health crisis	5.21 Proof of manager training in health crisis management<br><br>< 50 % (0)<br>OR<br>50-80 % (1)<br>OR<br>80 % + (2)			
			TOTAL Section E	/32
			E = (Total/32) X 100	E =

F. Health Research

- **Weighting: 5%**
  - Number of strategies: 5
  - Observable items: 8
- **Total score: 10**
  - Variation: 0-100%

STRATEGIES	OBSERVABLE ITEMS	POINTS	OBSERVATION	FINAL SCORE
S6.1 Research Ethics Board able to both conduct rigorous assessments and provide advice on clinical trials of new medications or treatments for the emerging pathogen while protecting participants	6.1 Research Ethics Board in the Network **(2)**			
S6.2 Capacity to transfer knowledge on emerging scientific data to the entire organization	6.2 Emerging scientific data knowledge transfer plan **(1)**			
	6.3 Proof of plan execution **(0.5)**<br>(Find proof of plan execution)			
	6.4 Dissemination tools available **(0.5)**			
S6.3 Presence of researchers with expertise in organizational research or social science methods to facilitate understanding of the impact of the crisis at the	6.5 Organizational researchers hired **OR** possibility of quickly mobilizing researchers through an academic partner **(2)**			

organization level and provide recommendations				
**S6.4 Multidisciplinary research team able to contribute to the development of policies, action plans and organizational strategies**	6.6 Multidisciplinary research committee to assess the status of the crisis and provide recommendations to the leadership team **(1)**			
	6.7 Proof of committee meetings **(1)**			
**S6.5 Research incentives**	6.8 Budget available for research development to improve both population health and organizational performance in times of crisis **(2)**			
			TOTAL Section F	/10
			F = (Total/10) X 100	**F =**

G. Ethics and Values

- **Weighting: 5%**
  - Number of strategies: 5

- Observable items: 6

➧ **Total score: 10**

- Variation: 0-100%

STRATEGIES	OBSERVABLE ITEMS	POINTS	OBSERVATION	FINAL SCORE
S7.1 Capacity to manage cancellation of non-urgent cases	7.1 Management plan for non-urgent clinical cases (2)			
S7.2 Monitoring of the impact of the crisis on more vulnerable individuals or groups	7.2 Committee for monitoring and analyzing the impact of the crisis on vulnerable populations and measures to cope with the crisis to ensure equity of access to care and access to information (2)<br><br>E.g., persons with disabilities, Indigenous peoples, visible minorities, seniors, etc.			
S7.3 Protection of employees' physical and mental health	7.3 Plan for protecting the physical and mental health of the Network's employees (1)			
	7.4 Proof of plan execution (1)			

S7.4 Taking the potential impacts on vulnerable populations (people with disabilities, Indigenous peoples, visible minorities, seniors, etc.) into consideration in clinical and administrative protocols, as well as proposed innovations, to mitigate inequalities and environmental impacts	7.5 Proof of analysis of clinical and organizational protocols by the committee responsible in the context of the crisis **(2)**			
S7.5 Analysis of the impact of social isolation on the most vulnerable populations, including employees and inpatients	7.6 Plan to mitigate the impact of social isolation on the most vulnerable populations **(2)**			
			TOTAL Section G	/10
			G = (Total/10) X 100	G =

H. Training

- **Weight: 10%**
  - Number of strategies: 6
  - Observable items: 6
- **Total score: 12**
  - Variation: 0-100%

STRATEGIES	OBSERVABLE ITEMS	POINTS	OBSERVATION	FINAL SCORE
S8.1 Internal training program for leaders on managing a health crisis more effectively (for example, emergency measures)	8.1 Availability of an internal training program for leaders in a crisis context (2)			
S8.2 Infection prevention and control training program for employees	8.2 Infection prevention and control training program (2)			
S8.3 Health crisis management training program for employees	8.3 Health crisis management training program for employees (2)			
S8.4 Easily configurable and adaptable distance training tool	8.4 Availability of an online education platform (2)			
S8.5 Capacity to develop educational strategies adapted to the target audience	8.5 Availability of education/pedagogy experts for creating			

	content adapted to the target audience **(2)**			
**S8.6 Capacity to provide appropriate training in the framework of applying redeployment plans**	8.6 Plan for training employees in the event of emergency redeployment **(2)**			
			TOTAL Section H	/12
			H = (Total/12) X 100	H =

Final weighting

Calculation of health crisis management capacity (HCMC)

1. Place the percentage calculated for each section in the “Score for sections A-H” column.
2. Perform the calculations in the “adjusted score” column. This calculation will adjust the totals to better reflect the weighted score for each section.
3. Calculate the sum of the “adjusted score” column. This result will provide the HCMC indicator.

Calculation chart

Dimension	Proposed weighting	Score for sections A-H (%)	Adjusted score
Clinical Care Management	20%	A =	A x 0.2 =
Resources	20%	B =	B x 0.2 =
Governance and Leadership	10%	C =	C x 0.1 =
Communication and Technologies	10%	D =	D x 0.1 =
Infection Prevention and Control	20%	E =	E x 0.2 =
Health Research	5%	F =	F x 0.05 =
Ethics and Values	5%	G =	G x 0.05 =
Training	10%	H =	H x 0.1 =
		FINAL SCORE (HCMC)<0}	HCMC =

HCMC formula	*HCMC (%) = (A x 0.2) + (B x 0.2) + (C x 0.1) + (D x 0.1) + (E x 0.2) + (F x 0.05) + (G x 0.05) + (H x 0.1)*

Notes

Breitner Gomes Chaves, MD, MPH, PhD

Breitner.GomesChaves@vitalitenb.ca
Research and Health evaluation unit

Vitalité Heath Network

Janvier 2023
